# Supplementary material for: Stardust: improving spatial transcriptomics data analysis through space-aware modularity optimization-based clustering
Source: Gigascience. 2022 Aug 10;11:giac075. doi: 10.1093/gigascience/giac075 (PMC9364686; doi:10.1093/gigascience/giac075)
Supplement: giac075_GIGA-D-21-00411_Original_Submission [file giac075_giga-d-21-00411_original_submission.pdf]

GigaScience

Stardust: improving spatial transcriptomics data analysis through space aware modularity optimization based clustering.

--Manuscript Draft--

Manuscript Number:	GIGA-D-21-00411
Full Title:	Stardust: improving spatial transcriptomics data analysis through space aware modularity optimization based clustering.
Article Type:	Technical Note
Funding Information:	JPcofuND2 Personalised Medicine for Neurodegenerative Diseases (JPND2019-466-037) — Prof. Rosalba Giugno
Abstract:	Background<br><br>Spatial transcriptomics (ST) combines stained tissue images with spatially resolved high-throughput RNA sequencing. The spatial transcriptomic analysis includes challenging tasks like clustering, where a partition among data points (spots) is defined by means of a similarity measure. Improving clustering results is a key factor as clustering affects subsequent downstream analysis. State-of-the-art approaches group data by taking into account transcriptional similarity and some by exploiting spatial information as well. However, it is not yet clear how much the spatial information combined with transcriptomics improves the clustering result.<br><br>Results<br><br>We propose a new clustering method, Stardust, that easily exploits the combination of space and transcriptomic information in the clustering procedure through a manual or fully automatic tuning of algorithm parameters. We evaluated Stardust results by analyzing ST datasets available on the 10X Genomics website and comparing clustering performances with state-of-the-art approaches by measuring the spots stability in the clusters. Stability is defined by the tendency of each point to remain clustered with the same neighbours when perturbations are applied.<br><br>Conclusions<br><br>Stardust is an easy-to-use methodology allowing to define how much spatial information should influence clustering on different tissues and achieving more stable results than state-of-the-art approaches.
Corresponding Author:	Rosalba Giugno<br>University of Verona<br>Verona, ITALY
Corresponding Author Secondary Information:	
Corresponding Author's Institution:	University of Verona
Corresponding Author's Secondary Institution:	
First Author:	Simone Avesani
First Author Secondary Information:	
Order of Authors:	Simone Avesani
	Eva Viesi
	Luca Alessandrì
	Giovanni Motterle
	Vincenzo Bonnici

Powered by Editorial Manager® and ProduXion Manager® from Aries Systems Corporation

	Marco Beccuti
	Raffaele Calogero
	Rosalba Giugno
	Vincenzo
	Bonnici4
**Order of Authors Secondary Information:**	
**Additional Information:**	
**Question**	**Response**
Are you submitting this manuscript to a special series or article collection?	No
**Experimental design and statistics**<br><br>Full details of the experimental design and statistical methods used should be given in the Methods section, as detailed in our Minimum Standards Reporting Checklist. Information essential to interpreting the data presented should be made available in the figure legends.<br><br>Have you included all the information requested in your manuscript?	Yes
**Resources**<br><br>A description of all resources used, including antibodies, cell lines, animals and software tools, with enough information to allow them to be uniquely identified, should be included in the Methods section. Authors are strongly encouraged to cite Research Resource Identifiers (RRIDs) for antibodies, model organisms and tools, where possible.<br><br>Have you included the information requested as detailed in our Minimum Standards Reporting Checklist?	Yes
**Availability of data and materials**<br><br>All datasets and code on which the conclusions of the paper rely must be	Yes

Powered by Editorial Manager® and ProduXion Manager® from Aries Systems Corporation

either included in your submission or deposited in publicly available repositories (where available and ethically appropriate), referencing such data using a unique identifier in the references and in the "Availability of Data and Materials" section of your manuscript.<br><br>Have you have met the above requirement as detailed in our Minimum Standards Reporting Checklist?	

Powered by Editorial Manager® and ProduXion Manager® from Aries Systems Corporation

Manuscript Click here to access/download;Manuscript;Stardust (1).pdf

Stardust: improving spatial transcriptomics data analysis through space aware modularity optimization based clustering.

Simone Avesani[1*], Eva Viesi[1*], Luca Alessandrì[2*], Giovanni Motterle[1], Vincenzo Bonnici[4], Marco Beccuti[3], Raffaele Calogero[2#], Rosalba Giugno[1#]

[1]Department of Computer Science, University of Verona, Verona, 37134, Italy,

[2]Department of Molecular Biotechnology and Health Sciences, University of Turin, Turin, 10126, Italy,

[3]Department of Computer Science, University of Turin, Turin, 10149, Italy,

[4]Department of Mathematical, Physical and Computer Sciences, University of Parma, Parma, 43121, Italy.

* equal contributor
# equal contributor

Abstract

Background: Spatial transcriptomics (ST) combines stained tissue images with spatially resolved high-throughput RNA sequencing. The spatial transcriptomic analysis includes challenging tasks like clustering, where a partition among data points (spots) is defined by means of a similarity measure. Improving clustering results is a key factor as clustering affects subsequent downstream analysis. State-of-the-art approaches group data by taking into account transcriptional similarity and some by exploiting spatial information as well. However, it is not yet clear how much the spatial information combined with transcriptomics improves the clustering result.
Results: We propose a new clustering method, *Stardust*, that easily exploits the combination of space and transcriptomic information in the clustering procedure through a manual or fully automatic tuning of algorithm parameters. We evaluated *Stardust* results by analyzing ST datasets available on the 10X Genomics website and comparing clustering performances with state-of-the-art approaches by measuring the spots stability in the clusters. Stability is defined by the tendency of each point to remain clustered with the same neighbours when perturbations are applied.
Conclusions *Stardust* is an easy-to-use methodology allowing to define how much spatial information should influence clustering on different tissues and achieving more stable results than state-of-the-art approaches.

Keywords: Spatial transcriptomics analysis; clustering; stability scores, parameters tuning, software comparison

Background

Single-cell RNA sequencing (scRNA-seq) has emerged as an essential tool to investigate cellular heterogeneity [1]. Individual cells of the same phenotype are commonly viewed as identical functional units of a tissue or organ. However, single-cell sequencing results suggest the presence of a complex organization of heterogeneous cell states producing together system-level functionalities. Thus, the comprehension of cell transcriptomics in their morphological context is crucial to understanding the effect of tissue organization in complex diseases, like specific cancer subtypes [2]. The pioneering technology called Spatial Transcriptomics (ST) [3,4,5] is able to preserve spatial information in transcriptomics, by integrating the features of microarray and the scRNA-seq barcoding system. In contrast to single-cell sequencing, spatial transcriptomics is only able to sequence the merged transcriptome profile of a small group of cells, also called a spot. By adding spatial information to scRNA-seq data, spatially resolved transcriptomes are reshaping our understanding of tissue functional

organization [6]. The progressive increase in the use of ST technology highlights the need for new methods for optimizing the extraction of knowledge from ST data [7,8,9,10].

Among all the emerging contributions in this young research area, several tools can be considered state of the art, mainly focused on cluster analysis downstream of ST data [11,12,13,14]. Pham et al. presented *stLearn* [11] to perform downstream analysis and cell types development states identification, by integrating tissue morphology, spatial dimensionality and the transcriptional information extracted from the cells. *stLearn* uses a deep neural network model to perform tile-based feature extraction from high-resolution histology images. The extracted morphological features, together with the expression value of the neighbouring spots, are exploited to smooth the gene expression data before the clustering task. Then, *stLearn* applies the Louvain or k-means clustering methods to derive the cluster to which each spot belongs. To cluster data, *stLearn* takes as input the number of principal components (PCs), the number of neighbours to build the kNN (k-nearest neighbors) graph and the resolution of the clustering algorithm.

In the same year, Hu et al. developed *SpaGCN* [12] which introduces a data integration approach based on graph convolution. *SpaGCN* as *stLearn* adds the histological information in the clustering task. It represents, through a weighted graph, the gene expression and also the similarity between each pair of spots. The latter is calculated taking into account the spatial coordinates of the spots and the average RGB value in a square of pixels to which the spots belong. The method allows increasing the weight given to histological information by varying the contribution of spots when aggregating gene expression data. To give a higher weight to images with a clear histological structure, the scaling parameter s can be increased when calculating the pairwise distance between spots. The hyper-parameter *l*, i.e. the characteristic length scale, can be tuned starting from the parameter *p*, which determines the percentage of total expression provided by the neighbours. The characteristic length scale determines the contribution of neighbouring spots when aggregating gene expression data by adjusting the edge weight between pairs of spots. Then, *SpaGCN* uses Louvain's method on the aggregated output matrix from graph convolution layers to perform clustering. In addition, this method enables setting the size of the RGB square of pixels, the number of PCs and the resolution of the clustering algorithm. Moreover, users have the possibility to discard or keep the image information by setting a Boolean flag. *SpaGCN*, as other tools, allows identifying spatially variable genes (SVGs) or meta genes for each resulting spatial domain to give a biological meaning to the detected clusters as reported in [7].

Subsequently, Dries et al. [13] presented *Giotto,* a toolbox of algorithms, including a Hidden Markov Random Field (HMRF) method, to analyze spatial gene expression profiling associated with histological images. *HMRF* is a graph-based model that characterizes how many spots are influenced by the neighbours in order to assign each spot to one of *k* spatial domains, i.e. clusters, where *k* is given in input by the user. In *Giotto*, the parameters to be set are the ones given in input to the *HMRF* function, that is, the expression values to use, the name of the spatial network employed, the spatially variable genes, the spatial dimensions, the name of dimension reduction method, the number of PCs, the number of spatial domains (or clusters), three parameters (beta, tolerance and z-score) for the initialization of the method. Differently from the above methods, *Giotto* uses only spatial information of the spots and not histological information.

The same direction of *Giotto* is followed in [14] where Zhao et al. proposed a method, called *BayesSpace*, based on a Bayesian statistical approach, that improves the identification of specific profiles in tissues by imposing a Markov random field (MRF) prior that gives higher weight to spots that are spatially close. It takes as input, the number of PCs and clusters, the spatial transcriptomic platform and a series of model parameters comprising the initial cluster assignments for spots or the method to obtain the initial assignments, the error model, the precision covariance structure, the number of MCMC (Markov chain Monte Carlo) iterations, the gamma smoothing parameter, the prior mean hyperparameter, the prior precision hyperparameter and the hyperparameters for Wishart distributed precision. *BayesSpace* allows to cluster the spots according to some a priori biological knowledge or otherwise using the elbow plot of the pseudo-log-likelihood to infer the number of clusters *q* that are given in input to the method. The authors show that *BayesSpace* outperforms, in terms of adjusted rand index and manual annotations, other methods in the literature, in particular, the three widely used non-spatial algorithms, namely k-means, mclust and Louvain's methods, and the two spatial clustering algorithms, *HMRF (Giotto)* and *stLearn*, on distinct samples of dorsolateral prefrontal cortex (DLPFC) dataset. This dataset was not analyzed in our comparisons due to the lack of publicly available reference manual annotation.

In this article, we propose a downstream ST analysis method, called *Stardust*, which takes into account both the expression and the physical location in the tissue section of the transcriptional profiles, to define the similarity of the objects to be grouped. With *Stardust*, we intend to investigate how much the spatial information combined with transcriptomics improves the clustering results. *Stardust* is based on the *Seurat* [15] algorithm for the clustering of scRNA-seq data which uses Louvain's method to perform clustering. By setting a parameter, the user can easily determine how much spatial information should affect the clustering similarity. Parameters can also be automatically derived from a tuning procedure. To understand how the usage of spatial information affects the stability of clusters, we evaluated both *Stardust* and *Seurat* on five publicly available 10x Genomics datasets, respectively derived from human breast cancer (HBC), mouse kidney (MK), human heart (HH) and human lymph node (HLN) tissues. We also compared *Stardust* with currently available ST clustering methods, including *stLearn, SpaGCN, Giotto* and *BayesSpace*. Each tool comes with specific parameters to be set by the user. We fully exploit such parameters. In order to assess clustering performances, alternatively from current contributions, which exploit functional aspects such as spatially variable genes [7], we defined two different objective clustering quality measures: the cell stability score (CSS) [16] and the coefficient of variation. The CSS defines the tendency of a cell or spot to remain clustered with the same group of elements when inducing a perturbation to the dataset, for instance by removing a random set of items, while the coefficient of variation value is derived from the CSS distribution as the ratio of the standard deviation to the mean, thus, a lower coefficient of variation means higher average stability and less variation from the mean. These comparison measures enable us to estimate the clustering stability of the different configurations and to assess whether considering spatial or morphological information leads to an improvement in terms of stability.

Results show that *Stardust* improves in a statistically significant manner the clustering stability by combining the transcriptional similarity of the spots with their spatial localization in several datasets with respect to *stLearn*, *SpaGCN* and *Giotto*, and it is comparable with *BayesSpace*. Furthermore, while other methods force spots to form misleading cluster structures, in which neighbouring spots are clustered without sensibly sharing their expression profile, *Stardust* appears to be unaffected by such behaviour. Finally, unlike other approaches, *Stardust* requires some parameters to be set by the user and their values can be assigned intuitively and automatically.

Methods

In this Section, we introduce the *Stardust* approach and the measures used for evaluating cluster performance. Datasets used for the clustering evaluation were downloaded from the 10X Genomics website, respectively derived from human breast cancer in two stages of disease (HBC1 and HBC2), mouse kidney (MK), human heart (HH) and human lymph node (HLN). For each dataset, we loaded the associated *Seurat* object and extracted the spot coordinates and the expression matrix. In order to reduce memory usage and computation time, we filtered in the data matrices the genes expressed in more than 10 spots. Preprocessed data, software code and tool documentation are available at https://github.com/InfOmics/stardust/.

Stardust

Stardust is implemented on top of the *Seurat* [15] clustering algorithm. *Seurat* package is one of the most used software for scRNAseq data analysis. *Seurat* implements a network-based clustering method called the Louvain algorithm [17] which encodes each element of a dataset as a node in a graph. Pairs of nodes are connected according to a pairwise measure of similarity based on the Euclidean distance between transcriptional profiles. Then, the algorithm performs a community detection step over the graph to retrieve the dataset partition. In *Stardust*, the distance matrix used in *Seurat* is replaced with a summation of two other matrices representing the transcriptional information and the spatial position of the spots.
The matrix regarding the transcriptional information is obtained from the pairwise Euclidean distance between transcriptional profiles in PCA space [18]. The matrix regarding the spatial position represents the pairwise spatial Euclidean distance between spots.

Given the distance matrix based on transcriptional profiles, T, and the distance matrix based on spot coordinates, S, a preliminary linear scaling step is applied to *S* in order to mitigate cases in which one measure overpowers the other. The scaling formula is the following one:

$$S' = S * \frac{max(T)}{max(S)} \quad (1)$$

where *max(T)* and *max(S)* are the maximum value in the matrices *T* and *S*, respectively. The final distance matrix *ST* is computed according to a user-defined parameter called space weight, a real number in [0,1], that defines how much to weigh the space with respect to the transcriptional similarity. *ST* is composed of a mixture of space and transcript information. The latter is always considered in its integrity, while space information is weighted by a scaling factor. The formula for *ST* is:

$$ST = S' * spaceWeight + T \tag{2}$$

Users configure a single parameter, i.e. *spaceWeight*, to control how much the space-based measure weights on the overall measure. The proposed methodology is very simple and flexible, indeed it can be incorporated into any existing single-cell clustering method. *Stardust*, developed as a standalone R package, can be easily installed with at least 4 cores and 8 GB RAM of computational resources from GitHub repository or used through the dedicated docker image.

Cluster Validation

In order to give a quantitative performance evaluation of the clustering obtained, we use two different clustering quality measures: the *cell stability score* (implemented in the rCASC package) [16] and the *coefficient of variation*. Finally, we investigate the *statistical validation* of the results.

Cell stability score. *rCASC* [16] takes as input a spatially resolved transcriptome and a clustering algorithm. It computes for each basic element of the dataset a *cell stability score (CSS)* that describes how much each element tends to remain clustered with the same other elements through a series of n repetitions of the clustering method on *n* different permutations of the dataset. The basic concept of the rCASC notion is that a good clustering should remain stable if a perturbation is applied to the dataset. A CSS is a real number in [0,1] associated with each individual spot in a dataset and computed running the following three steps. First, the desired clustering method is applied to the dataset and the cluster identity associated with each object - i.e. each spot in our application - are defined. Then, a subset of objects is removed from the original dataset (the percentage of objects is decided by the user) and is clustered. This step is repeated *n* times (*n* is a user parameter). We decided to set the number of permutations to 80 and to remove at each permutation 10% of the spots. In each of the repetitions, the percentage of spots that remain clustered with a particular spot in that permutation is determined by taking the results obtained in the first step as a reference. This value is stored. Finally, for each spot, it is computed how many times the set of spots clustered with it in each of the repetitions in the second step is equal to the set in the first step. This quantity is divided by the number of repetitions, obtaining the stability score. To reduce the computation time, we set a limit of 4 hours for the computation of the CSS for each tool configuration compared.

Coefficient of variation. To decide which configuration of *Stardust* was the best performing (based on stability scores) on a particular dataset with respect to the base *Seurat* clustering, we used the coefficient of variation defined as $\frac{\sigma}{|\mu|}$, where σ is the standard deviation of the distribution of the spot scores and μ is its mean. The lower the coefficient of variation, the better the performances. We also applied this metric to the other compared methods to evaluate the best performing configuration of each tool.

Statistical validation. We provide statistical evidence of the variation of cluster stability achieved by *Stardust* in the following way. Given a dataset we first apply *Stardust*, obtaining a set of stability scores i. Then, for 100 times, we shuffle the spots coordinates and re-apply *Stardust*, obtaining 100 sets of stability scores $j_1 \dots j_{100}$. For each couple (i,j_k) - with k in 1...100 - we evaluate the Wilcoxon statistical test with the null hypothesis that the distribution i is greater than j_k, i.e. the gain in stability obtained from the original spot coordinates is greater than the gain obtained from shuffled spot coordinates.

Results

In this Section, we assess the performance of *Stardust* on five datasets from 10x Genomics, respectively derived from human breast cancer in two stages of disease (HBC1 and HBC2), mouse kidney (MK), human heart (HH) and human lymph node (HLN).

To understand how the usage of spatial information affects the stability of clusters we computed the *cell stability scores* and evaluated the *coefficient of variation* of the stability scores (see Section Methods-Cluster Validation) of *Stardust* varying the parameter space weight (see Section *Methods*, formula (1)) and the clustering resolution. The space weights were set to 0, 0.25, 0.5, 0.75 and 1 and cluster resolution to 0.6, 0.8, and 1. space weight equals 0, when space is not considered, corresponds to comparing *Stardust* with respect to the transcriptomic based approach implemented by *Seurat*, here referred with terms *no space* used. space weight equals 1 means that space and transcripts have equal weight. The lower the coefficient of variation, the better the stability performances.

Results show that the introduction of spatial information (Figures 1 (a)) reduces the coefficient of variation of each *Stardust* configuration with respect to the configuration where space is not considered, i.e., the one corresponding to the *Seurat* implementation. Since setting the clustering parameters could be challenging, we also used the R package GenSA [19] solution to estimate space weight and clustering resolution maximizing the average cell stability score. We created a dedicated Docker image where GenSA runs the *Stardust* algorithm several times to tune all the required parameters. Coefficients of variation obtained from tuned parameters are shown in Figure 1 (a) with violet dots. To limit the computation time, *Stardust* tuning was run for each dataset, fixing to 10 the maximum number of GenSA iterations. Despite the low number of iterations, the estimated average cell stability scores are all higher or comparable with our best results. The achieved CVs confirmed the trends observed from the manual tests, allowing to explore *Stardust* configurations not considered before. Tuning running time varied from 4 to 24 hours, depending on many factors, including the dataset size and computational resources. By increasing the size of the datasets or the number of combinations of parameters, GenSA does not scale and therefore it is not straightforwardly applicable on the other ST algorithms that are far more complex than *Stardust* in terms of the entire set of parameters that can be configured.

Figures 1 (b) show cluster stability improvements by looking at five different *Stardust* space weight configurations and by keeping the cluster resolution fixed to 0.8, i.e. they focus the attention on one of the *cell stability score* distributions tested in Figures 1 (a). In all the datasets, space is able to increase the overall stability, and although this behaviour is not monotonous with the increasing of the weight given to the space, different space weights allow to achieve the best scores. Wilcoxon test (see Section *Methods-Cluster Validation*) was used to evaluate the significance of the results confirming that the increase of stability scores is not due to chance.

To complete *Stardust* evaluation, we compute the percentage of spots becoming stable or unstable (Figures 1 (c)) by comparing one of the best performing configurations of *Stardust* according to the coefficient of variation values (Figures 1 (a)) with the one not using space information for each dataset. Regardless of which threshold is used, the number of spots that become stable in *Stardust* with respect to the *no space* one is always more than the number of spots that become unstable for each dataset. However, from a cluster quality point of view, threshold values are reasonable if belonging in [0.5, 1], i.e. it is desired that each spot remains clustered with the same others in at least half of the rCASC permutations.

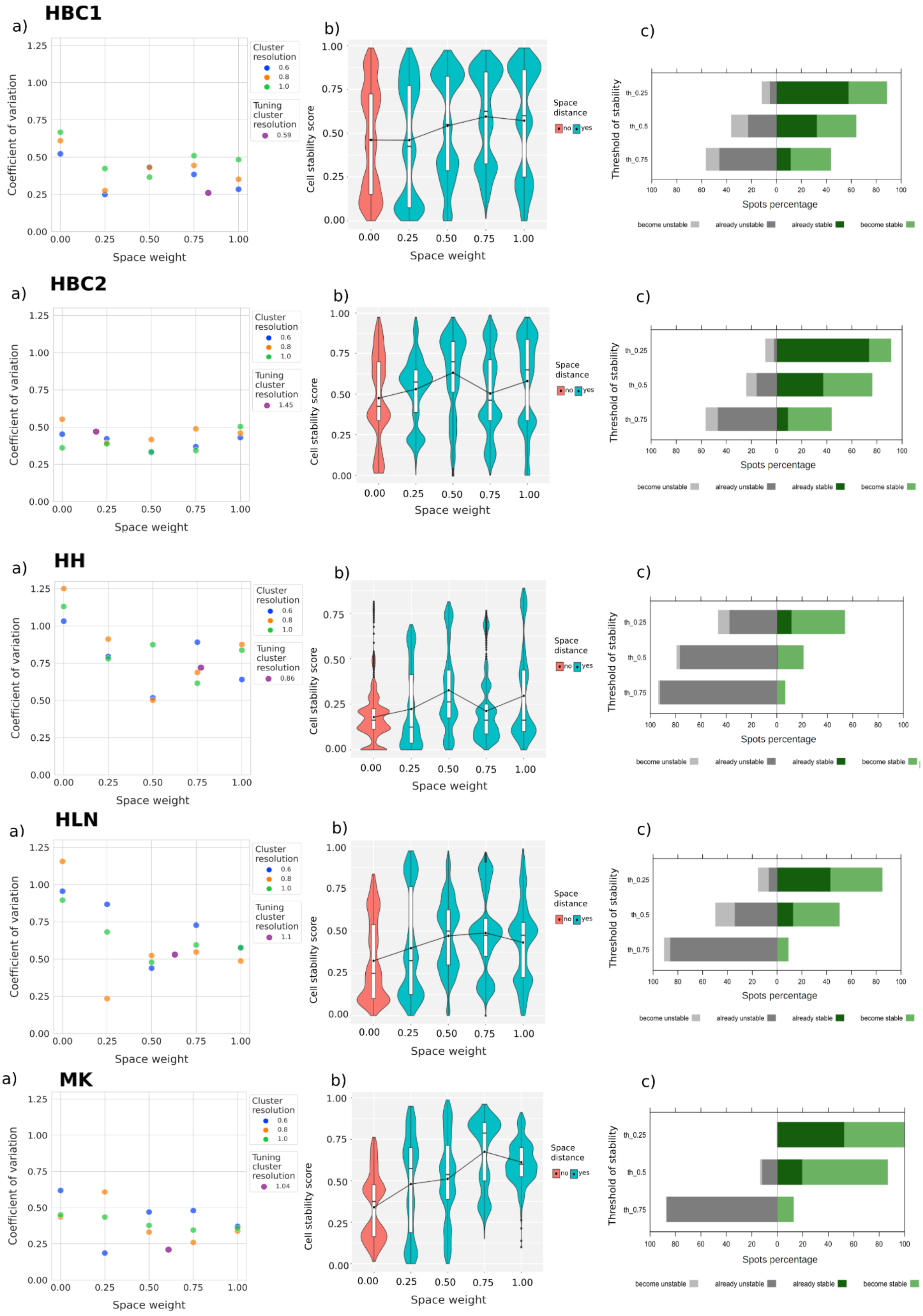

HBC1
HBC2
HH
HLN
MK
a)
b)
c)
Coefficient of variation
Space weight
Cluster resolution
0.6
0.8
1.0
Tuning cluster resolution
0.59
1.45
0.86
1.1
1.04
Cell stability score
Space distance
no
yes
Threshold of stability
th_0.25
th_0.5
th_0.75
Spots percentage
become unstable
already unstable
already stable
become stable

Figure 1: *Stardust* performance on five ST datasets: human breast cancer in two stages of disease (HBC1 and HBC2), mouse kidney (MK), human heart (HH) and human lymph node (HLN). (a) *Stardust* coefficient of variation for each configuration obtained varying the space weight and clustering resolution. Space weight and resolution tuned by maximizing the average cell stability score are shown with violet dots. (b) Stability scores comparison for 5 *Stardust* configurations with increasing space weight and cluster resolution fixed to 0.8. (c) The count of spots shifting from stable to unstable and vice versa at stability thresholds equal to 0.25, 0.5, 0.75, which set the limit to consider a spot stable (above the threshold) or unstable (below the threshold), comparing the best configuration of *Stardust* (i.e., with the lowest coefficient of variation) with the one not using space information.

Stardust was also tested and compared with state-of-the-art ST clustering methods, namely *stLearn, SpaGCN, Giotto* and *BayesSpace*, by analysing each individual 10x Genomics dataset. We evaluated *Stardust* stability scores with respect to the ones achieved with the other tools. We fixed the number of principal components to 10, which we found to be a good threshold for all the datasets analyzed through the '*elbow*' method proposed by rCASC [16]. The cluster resolution parameter was set to 0.6, 0.8 and 1 for each tool. For each resolution value, among the five configurations of *Stardust* obtained by varying the space weight parameter, we decided to represent the most stable ones, with space weight mainly equal to 0.25 and 0.5 for each dataset analyzed. Since *BayesSpace* and *Giotto* require a priori knowledge on the number of clusters, we derived it from the results of *Stardust* using for each cluster resolution the *Stardust* configuration with the lowest coefficient of variation score. Moreover, we tested *SpaGCN* both including and excluding histology image information.

The human breast cancer datasets HBC1 and HBC2 are composed of 3,798 and 3,987 spots, respectively. Concerning HBC1, by looking at the coefficient of variation in Figure 2 (a) and at the cell stability scores distributions in Figure 2 (b), *Stardust* and *stLearn* are the tools able to achieve the best clustering stability results and the highest average stability score. Figure 2 (b) shows the stability results of the compared tools, using their best configurations according to Figure 2 (a), i.e. the ones with the lowest coefficient of variation value: resolution 0.6 and space weight 0.25 for *Stardust*, resolution 0.6 and image True for *SpaGCN* and *stLearn*, resolution 0.8 for *BayesSpace* and resolution 0.6 for *Giotto*. *Stardust* reaches the lowest coefficients followed by *stLearn* and *BayesSpace*. Results for *SpaGCN* with cluster resolution 1 are missing because computation was out of a predefined time (> 4h).
Cluster results for a visual exploration together with the original tissue are shown in (Figure 2 (c)). According to the shifts of the stability scores (Figure 2 (d)), *Stardust and stLearn* best configurations show the highest percentage of spots becoming stable in the cluster assignments, followed by *BayesSpace* which is almost comparable with similar shifts scores.

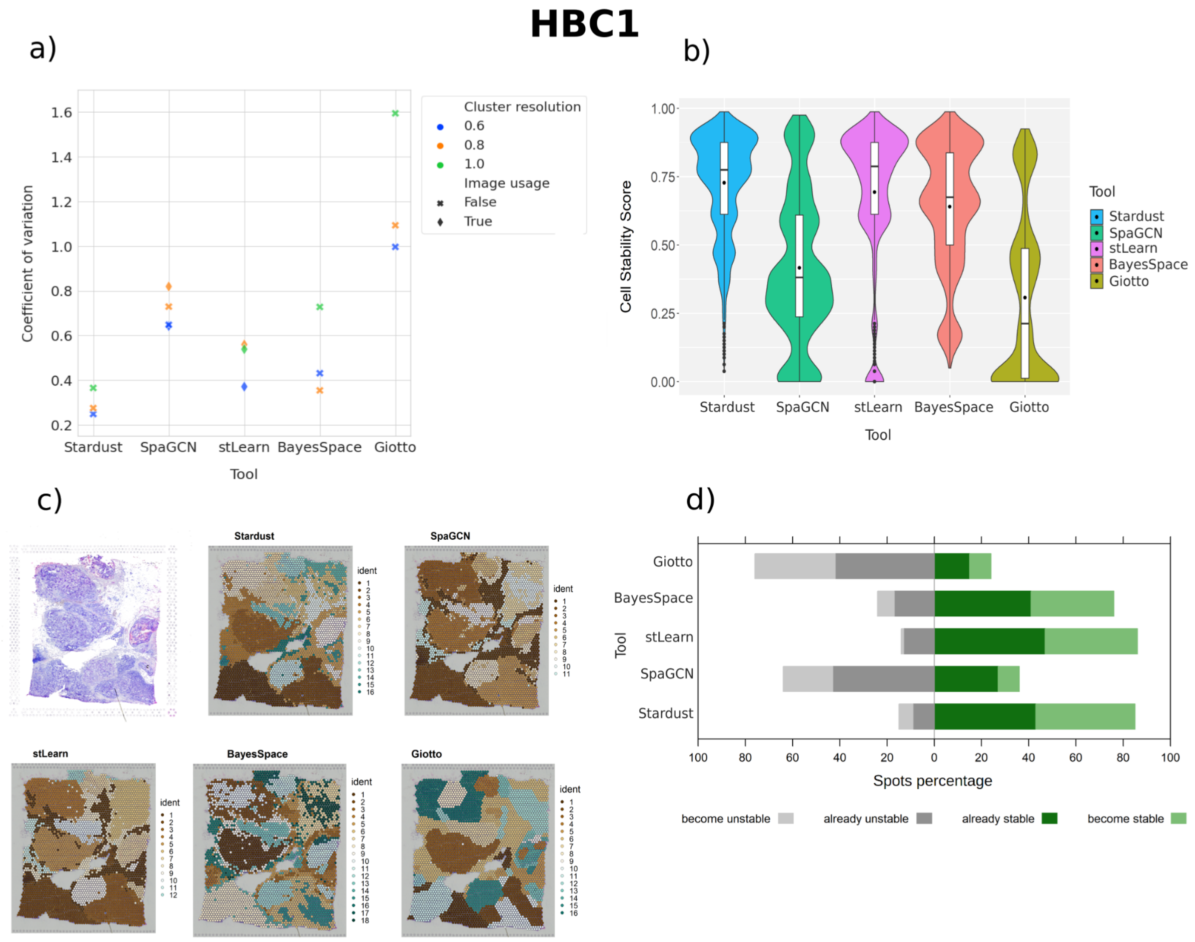

Figure 2: Comparison of *Stardust* and state of art tools on HBC1 dataset. (a) The coefficient of variation values derived from the stability score distribution of each tool configuration. The cluster resolution refers to the resolution parameter for the Louvain community detection algorithm, image usage tells whether the image is included in the clustering method. (b) The cell stability score distributions of the best performing configuration of each tool (i.e., the one with the lowest coefficient of variation). (c) The H&E (Hematoxylin & Eosin) stained tissue sample and a spatial plot for each best tool configuration with clusters of spots on the tissue section. (d) The stability scores shifts obtained comparing the best configuration of each tool with the base *Seurat* version, i.e., the one not considering space.

Analyzing HBC2, we observed that *Stardust* in terms of coefficient of variation (Figure 3 (a)) in some cases is comparable with *BayesSpace* and outperforms all other methods. The best configurations achieving the lowest coefficient of variation value are: resolution 0.6 and space weight 0.5 for *Stardust*, resolution 0.6 and image False for *SpaGCN*, resolution 1 for *stLearn* and *BayesSpace* and resolution 0.6 for *Giotto*. Results for *SpaGCN* with cluster resolution 0.8 without image and 1 are missing because computation was out of a predefined time (> 4h). *BayesSpace* outperforms all the other tools, including *Stardust,* in terms of average cell stability score (Figure 3 (b)). Cluster results for a visual exploration together with the original tissue are shown in (Figure 3 (c)). The analysis of shift scores (Figure 3 (d)) confirmed the clustering stability of *BayesSpace* followed by *Stardust* which show the highest percentage of spots becoming stable in the cluster assignments compared to the other methods.

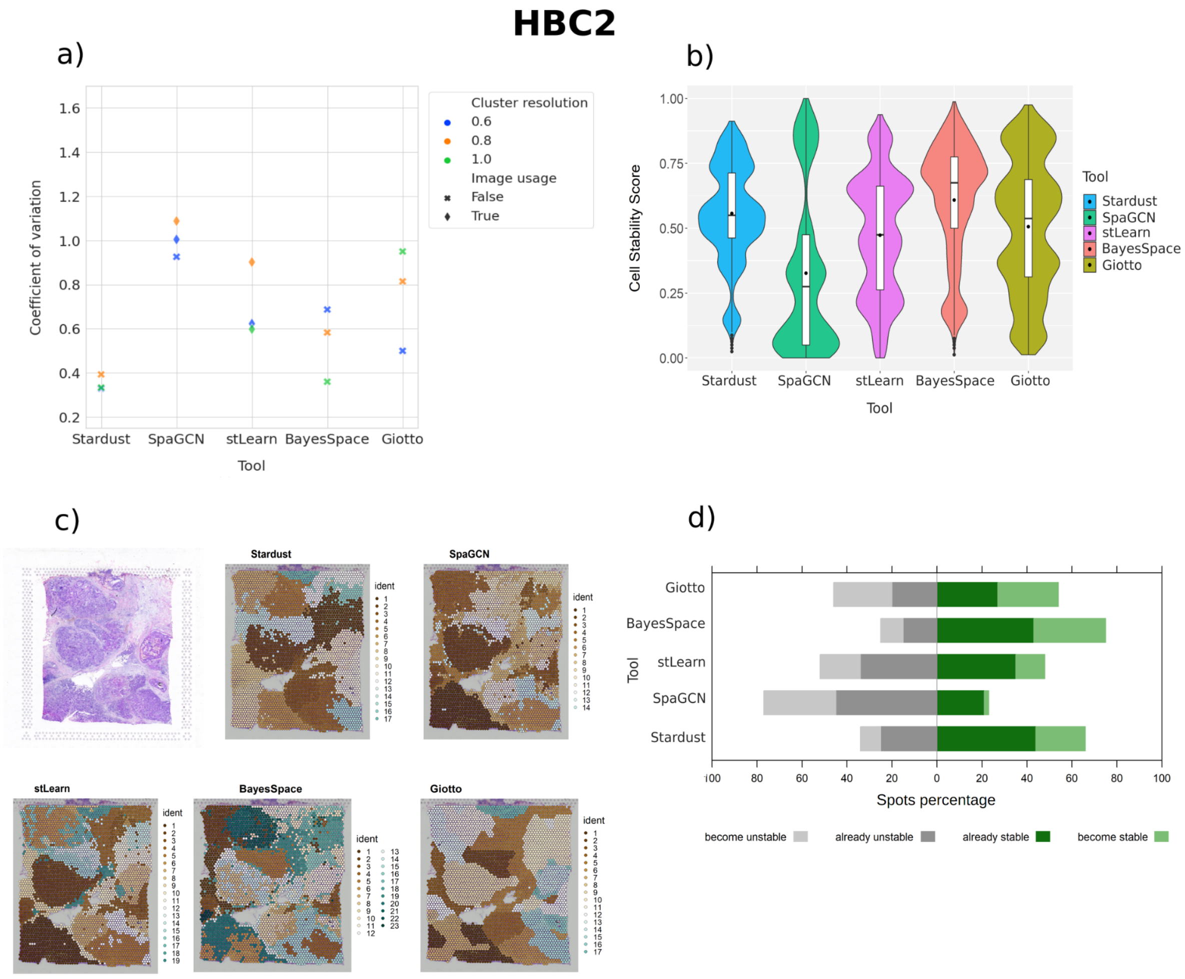

Figure 3: Comparison of *Stardust* and state of art tools on HBC2 dataset: (a) The coefficient of variation values derived from the stability score distribution of each tool configuration. The cluster resolution refers to the resolution parameter for the Louvain community detection algorithm, image usage tells whether the image is included in the clustering method. (b) The cell stability score distributions of the best performing configuration of each tool (i.e., the one with the lowest coefficient of variation). (c) The H&E (Hematoxylin & Eosin) stained tissue sample and a spatial plot for each best tool configuration with clusters of spots on the tissue section. (d) The stability scores shifts obtained comparing the best configuration of each tool with the base *Seurat* version, i.e., the one not considering space.

The Human Heart (HH) tissue is composed of 4,247 spots. In HH, analyzing the coefficients of variation (Figure 4 (a)), *Stardust* configurations achieve the lowest values overcoming the other tools. Overall, the best configurations achieving the lowest coefficient of variation value are: resolution 0.8 and space weight 0.5 for *Stardust*, resolution 0.6 and image False for *SpaGCN*, resolution 0.6 for *stLearn*, resolution 0.8 for *BayesSpace* and resolution 1 for *Giotto*. Result for *SpaGCN* with resolution 1 with the use of image is not reported because it is an out-of-scale value. Looking at the stability score distributions and at the score shifts, *BayesSpace* is the tool with the highest average stability (Figure 4 (b)) and the higher percentage of spots becoming stable (Figure 4 (d)), followed by *Stardust*. However, HH tissue demonstrates that *Stardust* is able to avoid finding deceptive structures when a well-defined histological pattern is not present (Figure 4 (c)). This tissue is characterized by a homogeneous architecture with respect to the morphological structure clearly visible in the other datasets analyzed. *Stardust*, as well as all the other tools, does not find a relevant number of spots showing a high degree of stability.

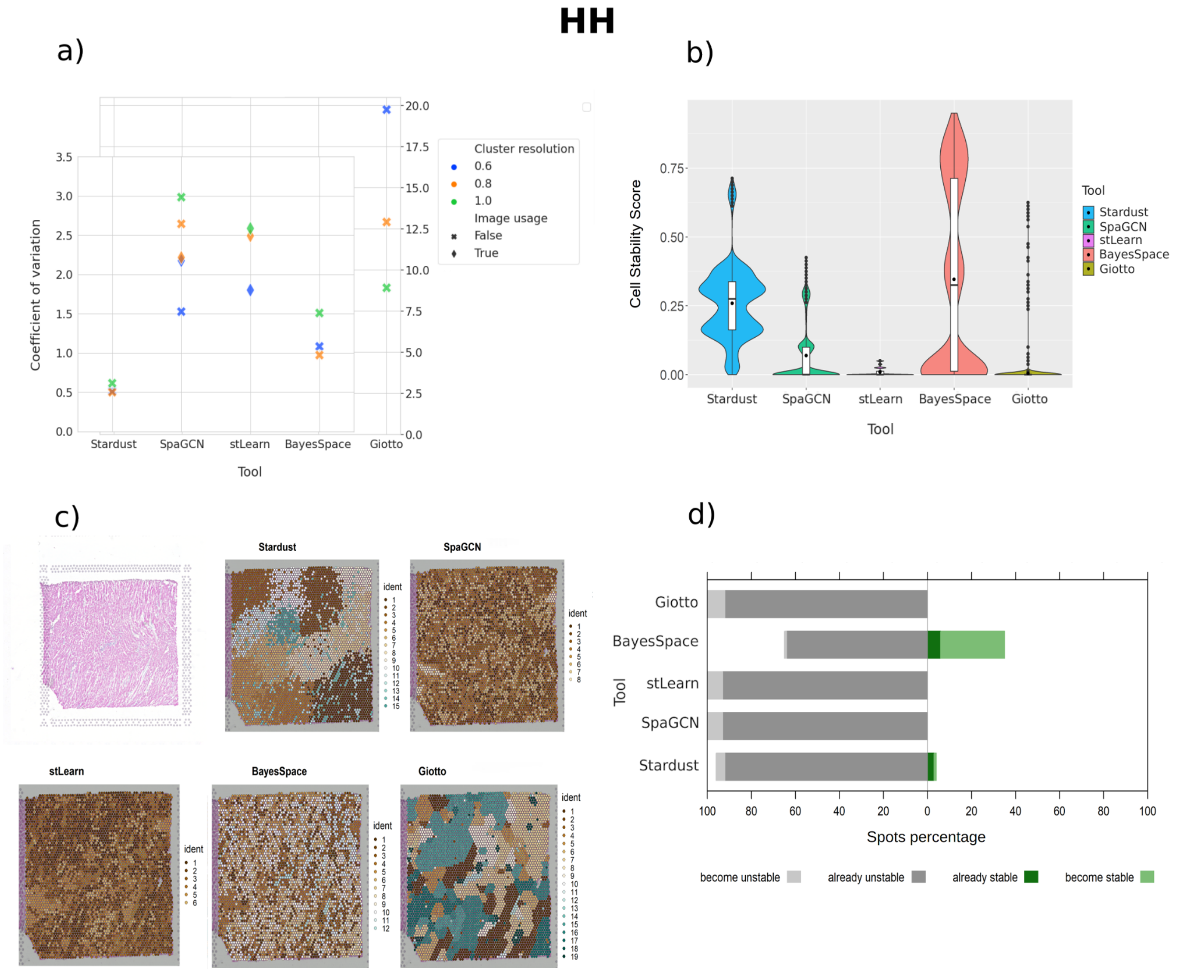

Figure 4: Comparison of *Stardust* and state of art tools on HH dataset. (a) The coefficient of variation values derived from the stability score distribution of each tool configuration. The cluster resolution refers to the resolution parameter for the Louvain community detection algorithm, image usage tells whether the image is included in the clustering method. (b) The cell stability score distributions of the best performing configuration of each tool (i.e., the one with the lowest coefficient of variation). (c) The H&E (Hematoxylin & Eosin) stained tissue sample and a spatial plot for each best tool configuration with clusters of spots on the tissue section. (d) The stability scores shifts obtained comparing the best configuration of each tool with the base *Seurat* version, i.e., the one not considering space.

The Human Lymph Node (HLN) dataset consists of 4,035 spots detected under the tissue. In HLN, *Stardust* is the tool that performs better in almost all its configurations according to the coefficient of variation values in Figure 5 (a). Overall, the best configurations achieving the lowest coefficient of variation value are: resolution 0.8 and space weight 0.25 for *Stardust*, resolution 0.6 and image False for *SpaGCN*, resolution 0.8 for *stLearn*, resolution 0.6 for *BayesSpace* and *Giotto*. Results for *SpaGCN* with cluster resolution 1 are not reported because computation was out of a predefined time (> 4h). *Stardust* exceeds the stability scores of the other tools (Figure 5 (b)). Cluster results for a visual exploration together with the original tissue are shown in (Figure 5 (c)). *BayesSpace* achieves good results, comparable to the *Stardust* ones while the other methods show very low stability scores, especially *Giotto*, in which there are no spots that become stable (Figure 5 (d)).

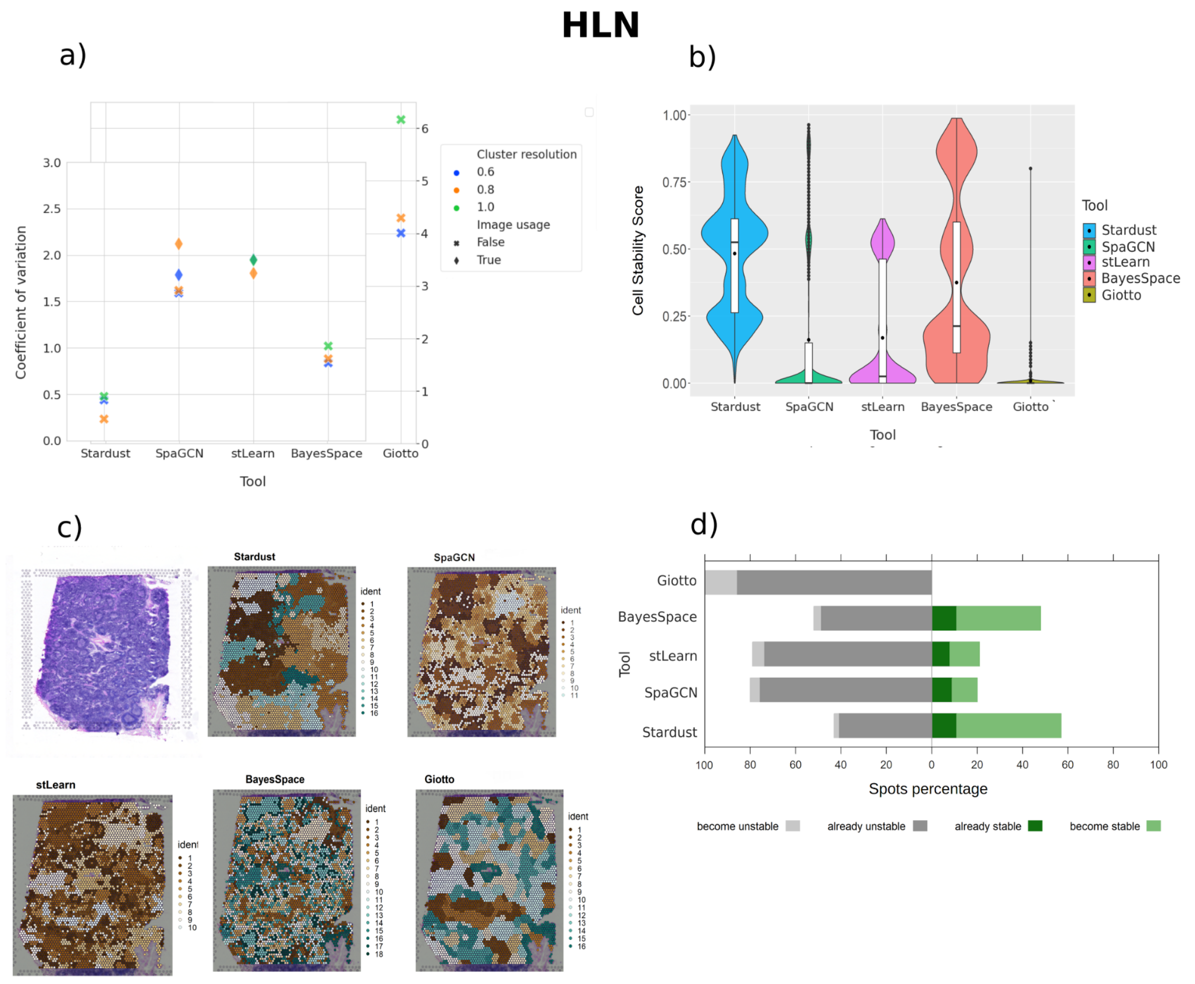

Figure 5: Comparison of *Stardust* and state of art tools on HLN dataset. (a) The coefficient of variation values derived from the stability score distribution of each tool configuration. The cluster resolution refers to the resolution parameter for the Louvain community detection algorithm, image usage tells whether the image is included in the clustering method. (b) The cell stability score distributions of the best performing configuration of each tool (i.e., the one with the lowest coefficient of variation). (c) The H&E (Hematoxylin & Eosin) stained tissue sample and a spatial plot for each best tool configuration with clusters of spots on the tissue section. (d) The stability scores shifts obtained comparing the best configuration of each tool with the base *Seurat* version, i.e., the one not considering space.

The Mouse Kidney (MK) dataset is composed of 1,438 spots. In MK, *Stardust* is the tool that clearly shows the lowest coefficient of variation value (Figure 6 (a)) and the highest stability scores, with an average value above 75% (Figure 6 (b)). The best configurations achieving the lowest coefficient of variation value are: resolution 0.6 and space weight 0.25 for *Stardust*, resolution 0.6 and image True for *SpaGCN*, resolution 0.8 for *stLearn*, resolution 1 for *BayesSpace* and resolution 0.6 for *Giotto*. Spatial clustering appears similar in almost all methods (Figure 6 (c)), except for *Giotto* for which the clustering stability is indeed significantly lower as compared to the other tools (Figure 6 (d)). *Stardust* overcomes all other tools in terms of the highest percentage of spots that become stable (Figure 6 (d)).

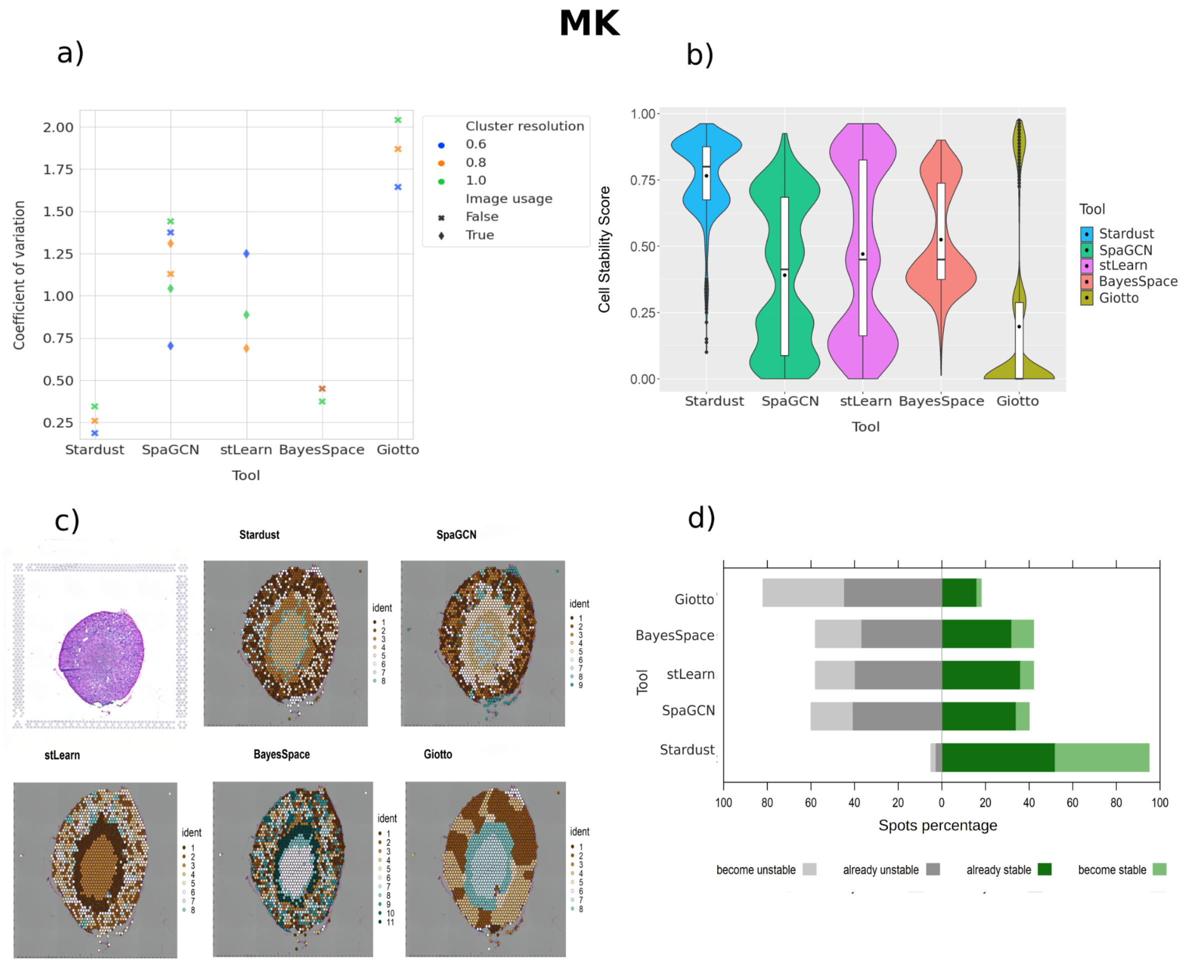

Figure 6: Comparison of *Stardust* and state of art tools on MK dataset. (a) The coefficient of variation values derived from the stability score distribution of each tool configuration. The cluster resolution refers to the resolution parameter for the Louvain community detection algorithm, image usage tells whether the image is included in the clustering method. (b) The cell stability score distributions of the best performing configuration of each tool (i.e., the one with the lowest coefficient of variation). (c) The H&E (Hematoxylin & Eosin) stained tissue sample and a spatial plot for each best tool configuration with clusters of spots on the tissue section. (d) The stability scores shifts obtained comparing the best configuration of each tool with the base *Seurat* version, i.e., the one not considering space.

Conclusion

We developed *Stardust,* an open-source and easy to install R package for Spatial Transcriptomics data clustering which integrates transcriptional and spatial information through a complete auto-tuned approach. The tool performances were evaluated by analysing the clustering stability through two stability measures: the cell stability score and the coefficient of variation. *Stardust* stability scores were compared with the ones achieved using *Seurat* to show how spatial information can significantly improve the clustering result. *Stardust* results were also compared with those achieved by the state-of-the-art tools investigated, including *BayesSpace*, *SpaGCN*, *stLearn* and *Giotto*. Results of each dataset analysis assess that *Stardust* achieves more stable results with respect to clustering performed without considering spatial information and also that it is a valid competitor, in terms of stability, to existing state-of-the-art clustering methods. Moreover, results demonstrated that the introduction of features from a histology image generally led to more unstable and misleading clustering results, particularly when the tissue section is quite uniform, and therefore, does not contain any particular structural information that could help clustering.

Availability of source code and requirements

- Project name: Stardust
- Project home page: https://github.com/InfOmics/stardust/, rCASC is available on https://github.com/InfOmics/rCASC and GenSA for Stardust is available on https://github.com/SimoneAvesani/Tuning_Stardust.
- Operating system(s): UNIX-like OS (MacOS or a Linux distribution)
- Programming language: R
- Other requirements: Docker
- License: MIT license
- Any restrictions to use by non-academics: None

Availability of supporting data

Datasets are available at https://github.com/InfOmics/stardust/. After the registration on the 10x Genomics website, each individual dataset can be downloaded from:

- Human breast cancer (HBC1): https://www.10xgenomics.com/resources/datasets/human-breast-cancer-block-a-section-1-1-standard-1-1-0
- Human breast cancer (HBC2): https://www.10xgenomics.com/resources/datasets/human-breast-cancer-block-a-section-2-1-standard-1-1-0
- Human heart (HH): https://www.10xgenomics.com/resources/datasets/human-heart-1-standard-1-1-0
- Human lymph node (HLN): https://www.10xgenomics.com/resources/datasets/human-lymph-node-1-standard-1-1-0
- Mouse kidney (MK): https://www.10xgenomics.com/resources/datasets/mouse-kidney-section-coronal-1-standard-1-1-0

Abbreviations

CSS: Cell Stability Score
DCIS: Ductal Carcinoma In Situ
DLPFC: Dorsolateral Prefrontal Cortex

GenSA: Generalized Simulated Annealing
HBC1: Human Breast Cancer 1
HBC2: Human Breast Cancer 2
HH: Human Heart
HLN: Human Lymph Node
HMRF: Hidden Markov Random Field
H&E: Hematoxylin & Eosin
IC: Invasive Carcinoma
KNN: K-Nearest Neighbor
MCMC: Markov chain Monte Carlo
MK: Mouse Kidney
MRF: Markov Random Field
PC: Principal Component
PCA: Principal Component Analysis
rCASC: reproducible Classification Analysis of Single Cell Sequencing Data
scRNA-seq: Single-cell RNA sequencing
ST: Spatial Transcriptomics

Author contributions

Conceptualization: RG and GM; Methodology: RG, SA, EV, LA, GM, VB, MB, RC; Supervision: RG, RC; Writing – review & editing: all; Code Writing: SA, EV, LA, GM; Test: SA, EV, LA, GM; Validation: RG, RC. None of the authors have any competing interests in the manuscript.

References

[1] Buettner, F., Natarajan, K. N., Casale, F. P., Proserpio, V., Scialdone, A., Theis, F. J., ... & Stegle, O. (2015). Computational analysis of cell-to-cell heterogeneity in single-cell RNA-sequencing data reveals hidden subpopulations of cells. *Nature biotechnology*, *33*(2), 155-160

[2] Lewis, S. M., Asselin-Labat, M. L., Nguyen, Q., Berthelet, J., Tan, X., Wimmer, V. C., ... & Naik, S. H. (2021). Spatial omics and multiplexed imaging to explore cancer biology. *Nature methods*, 1-16.

[3] Ståhl, P. L., Salmén, F., Vickovic, S., Lundmark, A., Navarro, J. F., Magnusson, J., ... & Frisén, J. (2016). Visualization and analysis of gene expression in tissue sections by spatial transcriptomics. *Science*, *353*(6294), 78-82.

[4] Asp, M., Bergenstråhle, J., & Lundeberg, J. (2020). Spatially resolved transcriptomes—next generation tools for tissue exploration. *BioEssays*, *42*(10), 1900221.

[5] Marx, V. (2021). Method of the Year: spatially resolved transcriptomics. *Nature Methods*, *18*(1), 9-14.

[6] Rao, A., Barkley, D., França, G. S., & Yanai, I. (2021). Exploring tissue architecture using spatial transcriptomics. *Nature*, *596*(7871), 211-220.

[7] Hu, J., Schroeder, A., Coleman, K., Chen, C., Auerbach, B. J., & Li, M. (2021). Statistical and machine learning methods for spatially resolved transcriptomics with histology. *Computational and Structural Biotechnology Journal*, *19*, 3829.

[8] Xu, Y., & McCord, R. P. (2021). CoSTA: unsupervised convolutional neural network learning for spatial transcriptomics analysis. *bioRxiv*.

[9] Teng, H., Yuan, Y., & Bar-Joseph, Z. (2021). Clustering spatial transcriptomics data. *Bioinformatics*.

[10] He, Y., Tang, X., Huang, J., Ren, J., Zhou, H., Chen, K., ... & Wang, X. (2021). ClusterMap for multi-scale clustering analysis of spatial gene expression. *Nature communications*, *12*(1), 1-13.

[11] Pham, D., Tan, X., Xu, J., Grice, L. F., Lam, P. Y., Raghubar, A., ... & Nguyen, Q. (2020). stLearn: integrating spatial location, tissue morphology and gene expression to find cell types, cell-cell interactions and spatial trajectories within undissociated tissues. *bioRxiv*.

[12] Hu, J., Li, X., Coleman, K., Schroeder, A., Irwin, D. J., Lee, E. B., ... & Li, M. (2020). Integrating gene expression, spatial location and histology to identify spatial domains and spatially variable genes by graph convolutional network. *bioRxiv*.

[13] Dries, R., Zhu, Q., Dong, R., Eng, C. H. L., Li, H., Liu, K., ... & Yuan, G. C. (2021). Giotto: a toolbox for integrative analysis and visualization of spatial expression data. *Genome biology*, *22*(1), 1-31.

[14] Zhao, E., Stone, M. R., Ren, X., Guenthoer, J., Smythe, K. S., Pulliam, T., ... & Gottardo, R. (2021). Spatial transcriptomics at subspot resolution with BayesSpace. *Nature Biotechnology*, 1-10.

[15] Butler, A., Hoffman, P., Smibert, P., Papalexi, E., & Satija, R. (2018). Integrating single-cell transcriptomic data across different conditions, technologies, and species. *Nature biotechnology*, *36*(5), 411-420.

[16] Alessandrì, L., Cordero, F., Beccuti, M., Arigoni, M., Olivero, M., Romano, G., ... & Calogero, R. A. (2019). rCASC: reproducible classification analysis of single-cell sequencing data. *Gigascience*, *8*(9), giz105.

[17] Blondel, V. D., Guillaume, J. L., Lambiotte, R., & Lefebvre, E. (2008). Fast unfolding of communities in large networks. *Journal of statistical mechanics: theory and experiment*, *2008*(10), P10008.

[18] Jolliffe, I. T., & Cadima, J. (2016). Principal component analysis: a review and recent developments. Philosophical Transactions of the Royal Society A: Mathematical, Physical and Engineering Sciences, 374(2065), 20150202.

[19] Xiang, Y., Gubian, S., Suomela, B., & Hoeng, J. (2013). Generalized simulated annealing for global optimization: the GenSA package. *R J.*, *5*(1), 13.

Supplementary Material

- Supplementary material file name : *Stardust_Supplementary_Section*
- Extension: *.pdf*
- Description: *Stardust tests*

Supplementary data of "Stardust: improving spatial transcriptomics data analysis through space aware modularity optimization based clustering."

Simone Avesani[1*], Eva Viesi[1*], Luca Alessandrì[2*], Giovanni Motterle[1], Vincenzo Bonnici[1], Marco Beccuti[3], Raffaele Calogero[2#], Rosalba Giugno[1#]

[1]Department of Computer Science, University of Verona, Verona, 37134, Italy,

[2]Department of Molecular Biotechnology and Health Sciences, University of Turin, Turin, 10126, Italy,

[3]Department of Computer Science, University of Turin, Turin, 10149, Italy.

[4]Dipartimento di Scienze Matematiche, Fisiche e Informatiche, University of Parma, Parma, 43121, Italy

* equal contributor
# equal contributor

Analysis of the influence of space information in clustering

In Figures S1-5 we depict how clusters are arranged in the 2D space of the tissue section and as space information influences the clusters in Mouse Kidney (MK), human lymph node (HLN), Human Heart (HH), and Human Breast Cancer (HBC1 and HBC2) datasets across the 5 *Stardust* configurations.

In Figures S1-5 (a) all points are displayed, while in Figures S1-5 (b) only points with stability scores greater than or equal to 0.5 are displayed. Score values are in [0, 1], so the threshold 0.5 means that in at least half of the permutations a spot remains clustered with the same other spots and can be considered a stable one.

Figure S1 showcases the true power of exploiting spatial information to achieve better stability scores when clustering Spatial Transcriptomic data. When space is not considered like in clusters plot with title space weight 0.0 almost none of the spots can achieve better stability scores than the threshold of 0.5. In the best *Stardust* configuration (space weight 1), the majority of them become stable.

An example, in particular, of increased spatial coherence in this dataset is represented by the area A circled in red in the tissue image in the Figure S1 (a) related to configuration space weight 0.0. Clusters 3 (spots depicted in white) and 6 (spots depicted in light brown) are located mainly in that area and are spatially close. They achieve poor stability scores as shown on the circled area C of the corresponding images in Figure S1 (b), due to high variability of cluster identity assignment to spots. *Stardust* collapses area A in one cluster (area B in Figure S1 (a)) that is spatially well-defined and achieves high stability scores (area D in Figure S1 (b)).

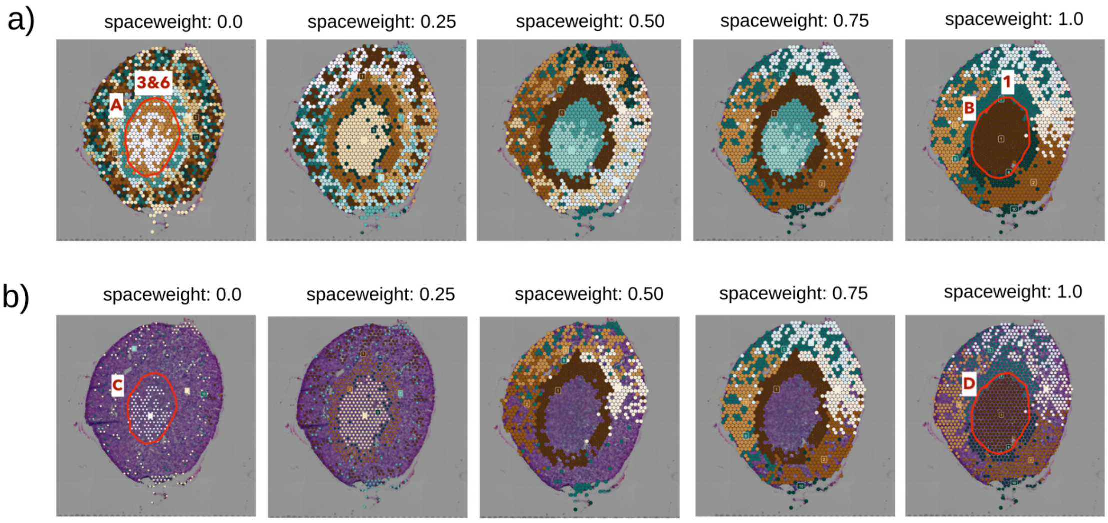

Figure S1: Spatial clusters plots in Mouse Kidney (MK) dataset. In (a) the results of 5 *Stardust* configurations with increasing space weight are shown. The same results, where only spots that have stability score >= 0.5 are visualized, are shown in (b). Each color corresponds to one of the 11, 9, 10, 10 and 10 cluster identities obtained in each configuration (in order of appearance), respectively.

In Figure S2 we show that the effect of introducing spatial information in the clustering task for the Human Lymph Node dataset, other than increasing the stability scores, is the mitigation of cases in which a subset of spots is not recognized as a unique cluster and is separated into two or more unstable clusters. An example of increased spatial coherence in this dataset is represented by the area A circled in red in the tissue image in Figure S2 (a) related to configuration space weight 0.0, i.e. when no space is used. Clusters 7 (spots depicted in white) and 9 (spots depicted in light cyan) are located mainly in that area and are spatially close. They achieve poor stability scores as shown on the circled area C of the corresponding images in Figure S2 (b), due to the high variability of cluster identity assignment to spots. The best performing *Stardust* configuration collapses area A in one cluster (area B in Figure S2 (a) configuration space weight 0.50) that is spatially well-defined and achieves high stability scores (area D in Figure S2 (b) on the same configuration).

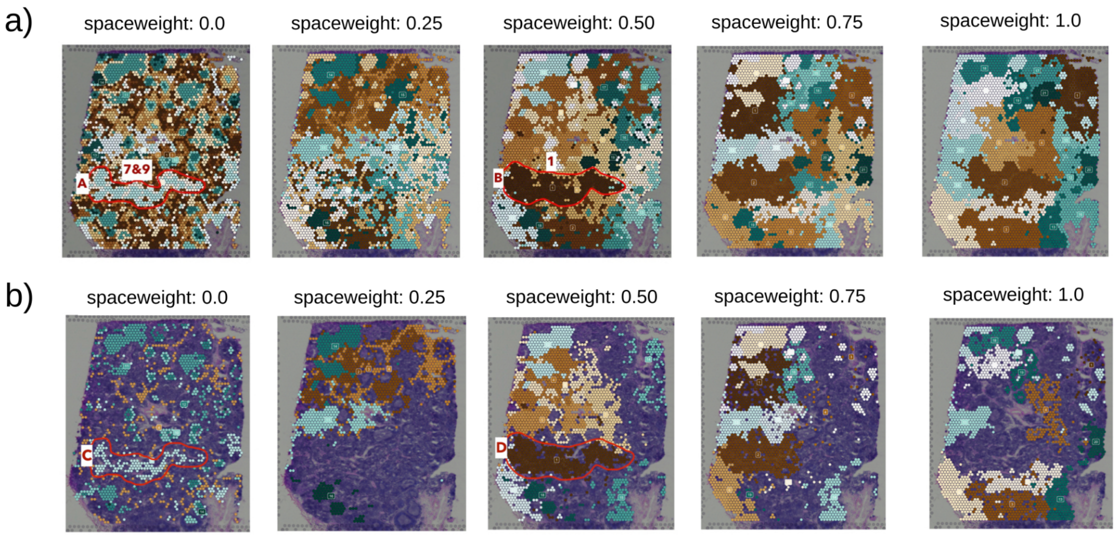

Figure S2: Spatial clusters plots in Human Lymph Node (HLN) dataset. In (a), the results of 5 *Stardust* configurations with increasing space weight are shown. The same results, where only spots that have stability score >= 0.5 are visualized, are shown in (b). Each color corresponds to one of the 14, 16, 19, 21 and 21 cluster identities obtained in each configuration (in order of appearance), respectively.

Visualizations in Figure S3 demonstrate that *Stardust* does not create a structure where it's not present. In fact, Human Heart (HH) tissue presents a similar architecture across the whole sample and the usage of space doesn't evidentiate a relevant number of stable subgroups of spots (clusters). The more evident results that *Stardust* is able to obtain are represented by the areas A and B circled in red in the tissue image in Figures S3 (a) and (b) related to configuration space weight 0.50. Although clusters 9 and 1 achieve good stability, they cover only a small portion of the tissue confirming that *Stardust* needs transcriptional variability across the tissue to identify structures in the tissue architecture.

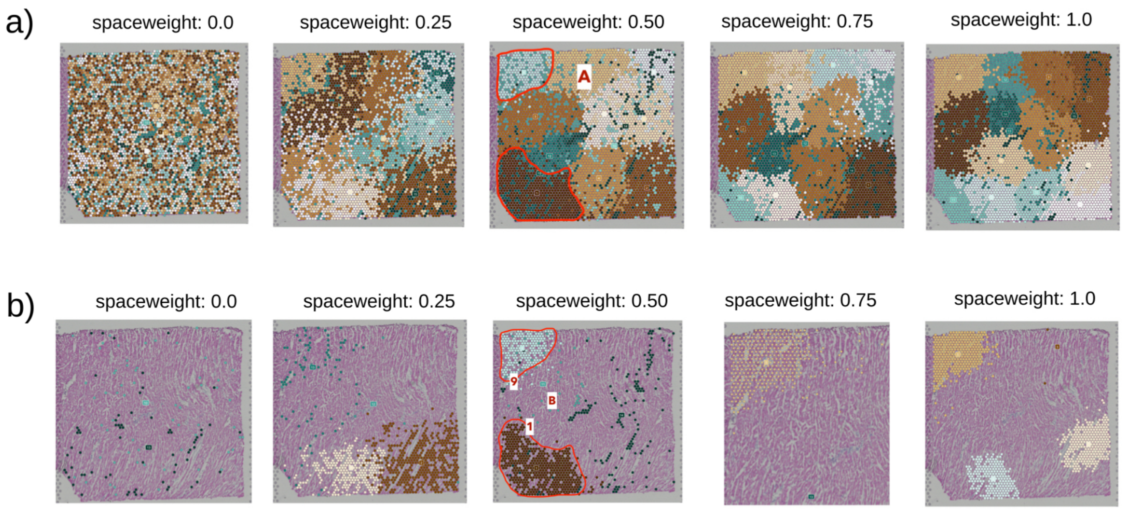

Figure S3: Spatial clusters plots in Human Heart (HH) dataset. In (a), the results of 5 *Stardust* configurations with increasing space weight are shown. The same results, where only spots that have stability score >= 0.5 are visualized, are shown in (b). Each color corresponds to one of the 13, 15, 14, and 15 cluster identities obtained in each configuration (in order of appearance), respectively.

In Figure S4, related to the Human Breast Cancer (HBC1) dataset, spatial information helps to mitigate cases in which distant spots are clustered together due to transcriptional similarity, but they do not belong to the same region in the tissue architecture. An example is represented by area A circled in red in the first cluster plot in Figure S4 (a). Area A contains the clusters 6 (spots in beige). When clusters 6 are considered as a whole as when space is not considered (i.e. configuration space weight 0.0), it achieves poor stability scores as shown on the circled area C (Figure S4 (b)). The best performing *Stardust* configuration (space weight 0.75) divides area A into two clusters (16, 14 in area B) that achieve high stability scores (area D). This is a notable result of *Stardust*, infact in [2] the authors report a manual annotation of the same tissue where the two clusters are identified as DCIS (ductal carcinoma in situ) compared to other clusters that have been defined as IC (invasive carcinoma). If the no use of space allows identifying the clusters 6 as a single cluster, these clusters are unstable (Figure S4 (b)) and therefore in unknown situations they would not be suggested to the pathologist to be noticed. Indeed, *Stardust* in its best configuration identifies them as very stable, albeit separate, clusters. The separation is foreseen as they are distant.

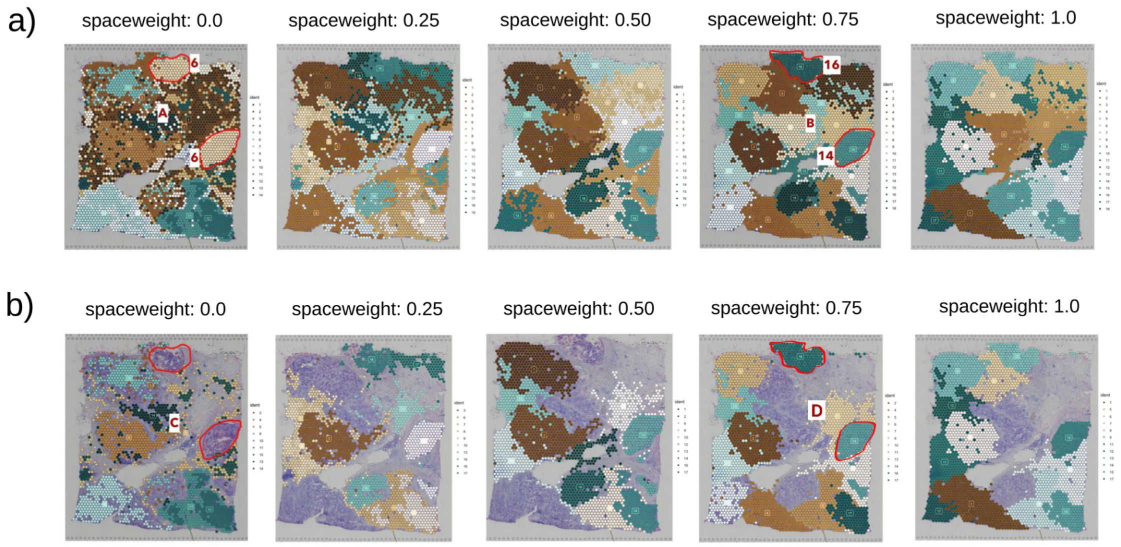

Figure S4: Spatial clusters plots in Human Breast Cancer (HBC1) dataset. In (a), the results of 5 *Stardust* configurations with increasing space weight are shown. The same results, where only spots that have stability score >= 0.5 are visualized, are shown in (b). Each color corresponds to one of the 14, 19, 17, 18 and 18 cluster identities obtained in each configuration (in order of appearance), respectively.

In Figure S5, related to the Human Breast Cancer (HBC2) dataset, spatial information increases the overall stability of two spatially neighbouring clusters. In this case, the strategy adopted by *Stardust* to increase stability scores is not to merge them as in HLN dataset but to reassign the cluster identity of each spot keeping two spatially neighbouring clusters. In the red circled area A in Figure S5 (a) when no space is used (i.e. configuration space weight 0.0) is depicted the original cluster arrangement that performs poorly (area C in Figure S5 (b)). In red circled area B (Figure S5 (a) configuration space weight 0.50) is shown the *Stardust* rearrangement with increased stability scores (area D in Figure S5 (b)).

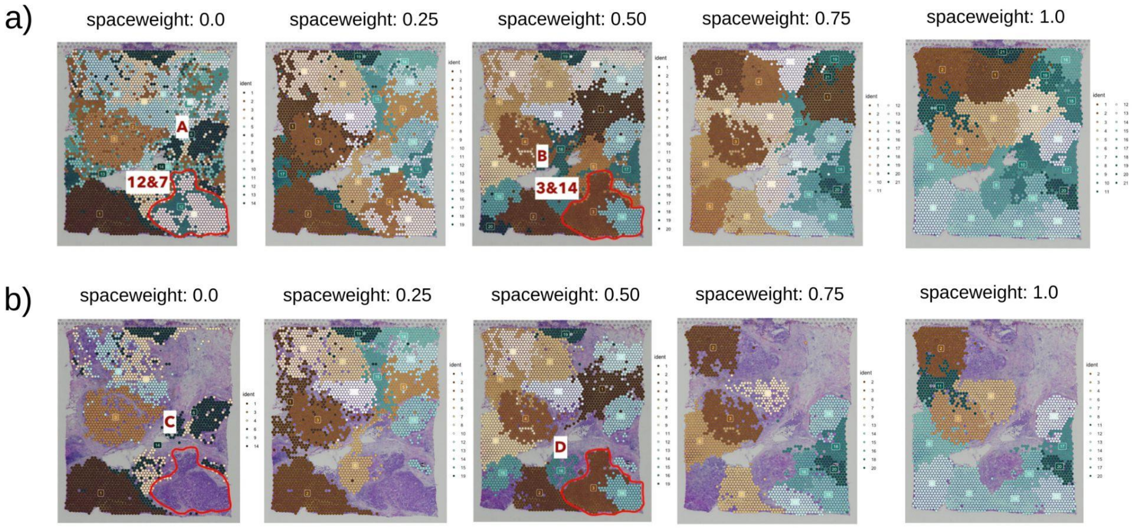

Figure S5: Spatial clusters plots in Human Breast Cancer (HBC2) dataset. In (a), the results of 5 *Stardust* configurations with increasing space weight are shown. The same results, where only spots that have stability score >= 0.5 are visualized, are shown in (b). Each colour corresponds to one of the 14, 19, 20, 21 and 21 cluster identities obtained in each configuration (in order of appearance), respectively.

Cover Letter Click here to access/download;Personal Cover;CoverLetter.pdf

UNIVERSITÀ di VERONA | Dipartimento di INFORMATICA

December 14th , 2021

Dear Editor,

We are pleased to submit our manuscript "*Stardust: improving spatial tranScripTomics data analysis through space awARe modularity optimization baseD clUSTering*" to be considered for publication in *Giga Science* as a *Technical Note*.

Our manuscript presents *Stardust*, a command-line tool to cluster Spatial transcriptomics profiles, i.e., focusing on a downstream analysis of 'big data' in the research area in biomedical sciences. Unlike existing methods, *Stardust* easily exploits the combination of space and transcriptomic information in the clustering procedure through a manual or fully automatic tuning of algorithm parameters. We evaluated *Stardust* results by analyzing ST datasets available on the 10X Genomics website and comparing clustering performances with state-of-the-art approaches by measuring the spots stability in the clusters. Stability is defined by the tendency of each point to remain clustered with the same neighbors when perturbations are applied. Therefore, *Stardust* is an easy-to-use methodology allowing to define how much spatial information should influence clustering on different tissues and achieving more stable results than state-of-the-art approaches. The source code and the documentation is available at https://github.com/InfOmics/stardust.

Finally, I declare that none of the authors have any potential competing interests; all authors have approved the manuscript for submission; the manuscript has not been published, or submitted for publication elsewhere.

Yours,

Rosalba Giugno, PhD

Dipartimento di Informatica
Strada le Grazie 15 - 37134 VERONA (IT) TEL. +39 045 2027071-7069
segreteria.di@ateneo.univr.it
P. IVA 01541040232 | C.F. 93009870234
