# Supplementary material for: Stardust: improving spatial transcriptomics data analysis through space-aware modularity optimization-based clustering
Source: Gigascience. 2022 Aug 10;11:giac075. doi: 10.1093/gigascience/giac075 (PMC9364686; doi:10.1093/gigascience/giac075)
Supplement: giac075_Response_to_Reviewer_Comments_Original_Submission [file giac075_response_to_reviewer_comments_original_submission.pdf]

# Author's Response To Reviewer Comments

Close

Dear Editor,

on the behalf of the other co-authors I enclose the revised manuscript entitled “Stardust: improving spatial transcriptomics data analysis through space aware modularity optimization based clustering”. We very much appreciated the reviewers’ comments and modified our manuscript accordingly. A list of all modifications according to the reviewers’ suggestions follows.

Best Regards
Rosalba Giugno

Replies to reviewer comments

Title: Stardust: improving spatial transcriptomics data analysis through space aware modularity optimization based clustering.
Authors: Simone Avesani, Eva Viesi, Luca Alessandrì, Giovanni Motterle, Vincenzo Bonnici, Marco Beccuti, Raffaele Calogero, Rosalba Giugno

We thank the reviewers for their comments which greatly helped us to improve the quality of the manuscript. Below are our answers to each comment and the corresponding modifications we did to the manuscript. We used the green colour to indicate in this document our answers to comments and the red colour in the manuscript and supplementary file to indicate parts that have been added or rewritten.

Reviewer #1: In this article the authors developed Stardust, a computational method that can be used for spatially-informed clustering by combining transcriptional profiles and spatial information. As spatial sequencing technologies gain popularity, it is important to develop tools that can efficiently process and analyse such datasets. Stardust is a new method that goes in this direction. It is particularly appealing to make use of the spatial information and relationships to cluster gene expression in these datasets.

Overall the quality of data used is high and the manuscript is clearly written. The algorithm behind Stardust is simple and consists of an interpolation between spatial and transcriptional distance matrices. A single parameter called space weight controls the contribution of the spatial distance matrix. The authors benchmark Stardust against other recently developed tools in five different spatial transcriptomics datasets by using two measures.

Stardust therefore holds the potential of being a useful method that can be applied in different datasets. Before recommending the manuscript for publication, however, the authors should thoroughly address the following points:

1. What is the rationale behind modelling the contributions as a linear sum of the spatial and transcriptional distance matrices? In particular, why did the authors not consider non-linear relationships as well? As cells neighboring in space often share similar transcriptional profiles (see for instance Nitzan et al., 2019 for this line of reasoning and several examples therein), I would expect product terms to be even more informative.

We thank the reviewer for this observation. According to his/her suggestions, we noticed that the product of the spatial and transcriptional distance matrices could lead to not desired clustering results. Indeed, such a model will potentially cluster together spots with a great transcriptomic distance but with a spatial distance close to 0.

Moreover, we decided not to apply the low reported in Nitzan et al., 2019 since the following plots show that spatial and transcriptional distances in 10x datasets do not follow the quadratic low reported in Nitzan et al., 2019.

Anyway, inspired by the reviewer's suggestion in the current revised manuscript we propose a new method called Stardust* that uses a nonlinear function to model space and expression as described below.
Concerning the linear sum of the spatial and transcriptional distance matrices, rather than using a fixed value for the space weight and having a linear model of the form: y = x + a*z, where x represents the vector of expression distances, z represents the vector of spatial distances and a is the space weight, we propose a new feature/method (called Stardust*) that uses a non-linear model of the form: y = x + w*z, where x represents the vector of expression distances, z represents the vector of spatial distances and w represents the vector of normalized expression distances. Thus, the spatial contribution depends on the expression distances distribution in the space. As implemented in Seurat, we consider that the expression data mirrors the tissue biology, but spatial contribution may help in adjusting clustering results. Moreover, both Stardust and Stardust* compute the distance among a pair of nodes on the vectors of the distances of each node to all other nodes. Finally, this new feature represents a parameter-free method concerning the spatial contribution in the clustering task. We refer to section Methods for a more detailed description, Results Section Figures 3-5 and Supplementary Figures S7-14, for performance evaluation. Stardust* outperformed compared methods and is comparable or better of Stardust. Figure S11 reports the scalability of Stardust, however, Stardust* has the same running time of Stardust therefore we refer to the same figure for scalability of Stardust*.

2. The authors demonstrate Stardust's performance only on datasets obtained with the 10X Visium platform. How does Stardust perform on higher-resolution methods, such as Slide-Seq, Seq-scope etc? As ST methods will improve in resolution in the future, it is critical to be able to analyze such datasets as well. An important question here concerns scalability: how well does Stardust scale with the number of cells/spots?

We thank the reviewer for this observation. We added the scalability of the proposed tool on different datasets from 10x Visium, Slide-seq and Seq-scope, (see Figure S11 in Supplementary material). Datasets differ in terms of number of spots.
Moreover, in Figures S12-4 in the Supplementary file we analyzed the Stardust* biological relevance of results by applying the Moran’s I on the Cerebellum Slide-seq dataset and on Colon and Liver Seq-scope datasets. For Seq-scope tests, we run tile-specific analysis, because the global distances between cells of the different contiguous tiles are not provided. We did not test our clustering tool on seqFISH and MERFISH datasets since they are probe-specific and up to now, they allow to characterize a limited number of RNA molecules (target thousands of single molecules with fluorescent probes).

3. In Fig. 1b conclusions are driven based on the CSS for different space weights, but only for a clustering parameter=0.8. What happens for other clustering values? And can the authors comment on why the different space weight values do not perform consistently across the datasets (i.e. 0.5 is better for HBC2 but 0.75 for MK)?

We thank the reviewer for this observation. The space weights vary among datasets since the spatial contribution strictly depends on the type of tissues the dataset represents. Using Stardust the user can run the tests by using his/her desiderata on the space weights. Moreover, the user can run the tuning tool for the automatically estimated optimal parameters, or use Stardust* which automatically and dynamically set the space weights according to the expression distances distribution in the space.

4. The authors compared Stardust with four other tools. The conclusion is that Stardust outperforms all other methods --and performs equivalently with BayesSpace. All of these methods, however, rely on choosing specific values for a number of parameters. Did the authors optimize these values when they benchmarked these methods against Stardust?

We thank the reviewer for this observation. Differently from Stardust and Stardust* the compared tools rely on more than a pair of parameters and on more complex models which make the tuning process a computational and intuitively hard problem. Therefore, we used the parameters suggested (default ones) by the developers, which correspond to those used by the authors in their original publication. Stardust* has only one parameter, which is the cluster resolution. We tuned all compared methods on cluster resolution and we show that Stardust* (which does not require other parameters to be set) outperforms all other tools run on the suggested author parameters.

5. I was able to successfully install Stardust and run it. The resulting clusters in the Seurat object, however, were all NAs. The authors should make an effort to better document how Stardust runs, including the input structure that the tool expects and potential issues that might arise.

We thank the reviewer for this observation. We are sorry about it. We have intensively tested the package on Linux based operating systems with at least 4 cores and 8 GB RAM of computational resources and a version of R > 4. All tests were successful. We are fully available to support the experience with Stardust and Stardust* of any user through the Github reporting issues system.

Reviewer #2: This work presents a new clustering method, Stardust, that has the potential to improve stability of clustering results against parameter changing. Stardust can assess the contribution to the clustering result by spatial information relative to gene expression information. Stardust appears to performs better than other methods in the two metrics used in this paper, stability and coefficient of variation. The essence of the method is the use of a spatial transcriptomics (ST) distance matrix as a simple linear combination of physical distance (S) and transcriptional distance (T) matrices. A weight factor is used for the S matrix to control and evaluate the contribution of the spatial information. The effort for evaluating multiple parameters and comparing with several latest methods and across a number of public spatial datasets is a highlight of the work. The authors also made the code available.

Major comments:

- The concept of combining spatial location and gene expression is not new and has been applied in most spatial clustering methods. It is not clear what are the new additions to current available methods, except for a feature to weigh the contribution of spatial components to clustering results.

We thank the reviewer for this observation. A spatial transcriptomics analysis involves upstream analysis such as data preprocessing, gene imputation and spatial decomposition, and downstream analysis such as spatial clustering, identification of spatial variable genes and genes/cells interactions. The technology is continuously evolving, raising significant challenges on the above workflow in all different steps, however, downstream analysis tends to be technology agnostic. Our proposed method fits on the downstream task to perform clustering on gene count and spot location matrices. It is known that clustering on its own is a complex task more on the aspect of interpretation of results than algorithm design; intuitive and simple methods such as k-means have been widely used for decades on large different domains. In this revised manuscript we further improved the contribution, we propose a simple but effective spatial cluster method with different properties. It allows users to explore the contribution of spatial clustering by setting a desiderata spatial weight, by testing several spatial weights, by automatically tuning the best weight, or by using a dynamic non-linear model that changes the weight in base of transcriptomics values of the surrounding space. Moreover, it can easily be integrated into spatial transcriptomics workflow analysis since the non-linear model is parameter-free; users need to set only the cluster resolution. Tests show that the proposed methods are scalable, effective and outperform existing methods. Moreover, we show by using Moran's I index that results maintain biological significance.

- The approach to assess the contribution of spatial information, by varying the weight factor from 0 to 1 is rather simple, because the contribution can be nonlinear and vary between spots/cells (e.g. spatial distance becomes more important for spots/cells that are nearer to each other; some genes are more spatially variable than the others; applying one weight factors for all genes and all spots would miss these variation sources)

We thank the reviewer for this observation. Inspired by the reviewer's suggestion in the current revised manuscript we also propose a method called Stardust* that uses a non-linear function to model space and expression as described below.
Concerning the linear sum of the spatial and transcriptional distance matrices, rather than using a fixed value for the space weight and having a linear model of the form: $y = x + a*z$, where x represents the vector of expression distances, z represents the vector of spatial distances and a is the space weight, Stardust* uses a non-linear model of the form: $y = x + w*z$, where x represents the vector of expression distances, z represents the vector of spatial distances and w represents the vector of normalized expression distances. Thus, the spatial contribution depends on the expression distances distribution in the space. As implemented in Seurat, we consider that the expression data mirrors the tissue biology, but spatial contribution may help in adjusting clustering results. Moreover, both Stardust and Stardust* compute the distance among a pair of nodes on the vectors of the distances of each node

to all other nodes. Finally, this new feature represents a parameter-free method concerning the spatial contribution in the clustering task.
We refer to section Methods for a more detailed description, Results Section Figures 3-5 and Supplementary Figures S7-14, for performance evaluation. Stardust* outperformed compared methods and is comparable or better of Stardust. Figure S11 reports the scalability of Stardust, however, Stardust* has the same running time of Stardust therefore we refer to the same figure for scalability of Stardust*.

- The 5 weight factors 0, 0.25, 0.50, 0.75, and 1 were used. However, this range of parameters provided too few data points to assess the impact of spatial factor. As seen in figures, the 5 data points do not strongly suggest a point where the spatial contribution is maximum/minimum due to large fluctuation of values in the y-axis.

We thank the reviewer for this observation. We used a few weight factors to show that users can investigate using his/hers desiderata weights which could be the best spatial contribution for each type of tissue. To assess the impact of spatial factors we applied an automatic tuning procedure to identify the best weight and resolution factors for each dataset to analyze. Moreover, in this revised version of the manuscript, we also implemented a new method which uses spatial information with no a priori assumption on its contribution, but simply following the expression distribution values across space. It dynamically changes the spatial factors according to the transcriptions values of the space.

- Although two performance metrics are used (stability and variation), there needs to be an additional metric about how the clustering results represent biological ground truth cell type composition or tissue architecture (for example, by comparing to pathological annotation). Consequently, it is unclear if the stardust results are closer to the biological ground truth or not.

We thank the reviewer for this observation. We added Figures 4, S6 and S12-4 showing that Stardust*'s results are biologically sound. By using the Moran's I index we demonstrate that genes in the top 100 genes with highest Moran Index are spatially correlated in accordance with the cluster results. Moreover, we show that clusters on HBC1 are consistent with pathologists' annotation using the annotation provided by Lewis et al. 2021 (see Figure 6) and that Slide-seq and Seq-scope clusters are consistent with the pre-annotated clusters provided in [18, 21] .

- Stardust was tested on multiple 10x Visium datasets, but different types of spatial transcriptomics data like seqFISH, Slideseq, MERFISH, ect. are also common. Extended assessment of potential applications to other technologies would be useful.

We thank the reviewer for this observation. We added the scalability of the proposed tool on different dimensions datasets from 10x Visium, Slide-seq and Seq-scope (see Figure S11 in Supplementary material).
Moreover, in Figures S12-4 in the Supplementary file we analyzed the Stardust* biological relevance of results by applying the Moran's I on the Cerebellum Slide-seq dataset and on Colon and Liver Seq-scope datasets. For Seq-scop tests, we run tile-specific analysis, because the global distances between cells of the different contiguous tiles are not provided. We did not test our clustering tool on seqFISH and MERFISH datasets since they are probe-specific and up to now, they allow to characterize a limited number of RNA molecules (target thousands of single molecules with fluorescent probes).

Minor comments:

- The paragraphs and figure legends in the Result section are repetitive.
- The result section is descriptive and there is no Discussion section.

We thank the reviewer for these observations. We have restructured the Result Section moving contents to the Supplementary materials and adding commentary text.

Close
