# Supplementary material for: Stardust: improving spatial transcriptomics data analysis through space-aware modularity optimization-based clustering
Source: Gigascience. 2022 Aug 10;11:giac075. doi: 10.1093/gigascience/giac075 (PMC9364686; doi:10.1093/gigascience/giac075)
Supplement: giac075_Response_to_Reviewer_Comments_Revision_1 [file giac075_response_to_reviewer_comments_revision_1.pdf]

Author's Response To Reviewer Comments

Close

Dear Editors,

We thank you and the reviewers for the valuable comments provided. In the attached response letter, we address these comments in a point-by-point manner. We are looking forward to hearing from you.

Yours sincerely,

Rosalba Giugno

Replies to reviewer comment

Title: Stardust: improving spatial transcriptomics data analysis through space aware modularity optimization based clustering.
Authors: Simone Avesani, Eva Viesi, Luca Alessandrì, Giovanni Motterle, Vincenzo Bonnici, Marco Beccuti, Raffaele Calogero, Rosalba Giugno

Reviewer #1: The authors have successfully addressed all raised points. The introduction of Stardust*, in particular, is a valuable enhancement of the method. Therefore, I recommend the manuscript for publication.

We are very thankful to the reviewer for the comment.

Reviewer #2: The authors have improved the initial manuscript markedly. There are a couple of important points regarding comparisons between Stardust and Stardust* that need to be addressed:

1. In which cases Stardust* improves over Stardust? It seems the results would be dependent on different biological systems (i.e., tissue types). The authors suggest both versions produce comparable results, but given the major change in the formula (replacing a constant weight with variable weights as normalised gene expression values to [0,1] minmax scale), there are likely differences between Stardust and Stardust*. For example, certain genes will have higher weight than the others, therefore making the effects of the weights variable among genes. For this example, the authors may assess highly abundant genes vs low abundant genes.

We are very thankful to the reviewer for the comment. We observed that generally Stardust* and Stardust are almost comparable in terms of clustering stability (see Figures S7-10 (d)) that report the number of spots becoming stable during the clustering task). There is no a priori criterium to assess whether one approach is better than the other with respect to the biological system under investigation. We suggest using Stardust if the user wants to decide a priori which weight to give to the spatial component or to perform an investigation on the most suited weight to be set for the analyzed tissue. Alternatively, we recommend using Stardust*, a parameter-free approach that gives comparable clustering results in terms of stability scores and does not require any additional effort by the user.

We are sorry, we forgot to clearly remark that to account for the bias in the difference between high and low abundant genes we used a pre-processing and normalization approach provided by the Seurat package [Hafemeister, C., Satija, R., 2019], called sctransform, for both Stardust and Stardust*. Moreover, we applied the principal component analysis to identify the highly variable genes that are used to compute the euclidean distances between the gene expression profiles.

2. In cases where spatial distances are important, Stardust* could be less accurate than Stardust

version with a high space weight. How Stardust* considers cases that spatial distance is as important as gene expression.

We thank the reviewer for the comment. In this case, we recommend using Stardust with space weight equal to 1, which corresponds to giving the same importance to the transcriptomic and spatial components. However, results showed that giving the same importance to the spatial component did not improve clustering stability.

Close
