# Supplementary material for: Stardust: improving spatial transcriptomics data analysis through space-aware modularity optimization-based clustering
Source: Gigascience. 2022 Aug 10;11:giac075. doi: 10.1093/gigascience/giac075 (PMC9364686; doi:10.1093/gigascience/giac075)
Supplement: giac075_Response_to_Reviewer_Comments_Revision_2 [file giac075_response_to_reviewer_comments_revision_2.pdf]

Author's Response To Reviewer Comments

Close

Dear Editors,

We thank you for the precision and attention with which you are following our work. We apologize for the delay in submitting it but we have been busy organizing a Bioinformatics conference on site.

Yours sincerely,

Rosalba Giugno

Close
