# Supplementary material for: Stardust: improving spatial transcriptomics data analysis through space-aware modularity optimization-based clustering
Source: Gigascience. 2022 Aug 10;11:giac075. doi: 10.1093/gigascience/giac075 (PMC9364686; doi:10.1093/gigascience/giac075)
Supplement: giac075_Reviewer_1_Report_Original_Submission — Nikos Karaiskos -- 1/15/2022 Reviewed [file giac075_reviewer_1_report_original_submission.pdf]

Reviewer Report

Title: Stardust: improving spatial transcriptomics data analysis through space aware modularity optimization based clustering.

Version: Original Submission **Date:** 1/15/2022

Reviewer name: Nikos Karaiskos

Reviewer Comments to Author:

In this article the authors developed Stardust, a computational method that can be used for spatially-informed clustering by combining transcriptional profiles and spatial information. As spatial sequencing technologies gain popularity, it is important to develop tools that can efficiently process and analyse such datasets. Stardust is a new method that goes in this direction. It is particularly appealing to make use of the spatial information and relationships to cluster gene expression in these datasets.

Overall the quality of data used is high and the manuscript is clearly written. The algorithm behind Stardust is simple and consists of an interpolation between spatial and transcriptional distance matrices. A single parameter called space weight controls the contribution of the spatial distance matrix. The authors benchmark Stardust against other recently developed tools in five different spatial transcriptomics datasets by using two measures.

Stardust therefore holds the potential of being a useful method that can be applied in different datasets. Before recommending the manuscript for publication, however, the authors should thoroughly address the following points:

1. What is the rationale behind modelling the contributions as a linear sum of the spatial and transcriptional distance matrices? In particular, why did the authors not consider non-linear relationships as well? As cells neighboring in space often share similar transcriptional profiles (see for instance Nitzan et al., 2019 for this line of reasoning and several examples therein), I would expect product terms to be even more informative.
2. The authors demonstrate Stardust's performance only on datasets obtained with the 10X Visium platform. How does Stardust perform on higher-resolution methods, such as Slide-Seq, Seq-scope etc? As ST methods will improve in resolution in the future, it is critical to be able to analyze such datasets as well. An important question here concerns scalability: how well does Stardust scale with the number of cells/spots?
3. In Fig. 1b conclusions are driven based on the CSS for different space weights, but only for a clustering parameter=0.8. What happens for other clustering values? And can the authors comment on why the different space weight values do not perform consistently across the datasets (i.e. 0.5 is better for HBC2 but 0.75 for MK)?
4. The authors compared Stardust with four other tools. The conclusion is that Stardust outperforms all other methods --and performs equivalently with BayesSpace. All of these methods, however, rely on choosing specific values for a number of parameters. Did the authors optimize these values when they benchmarked these methods against Stardust?
5. I was able to successfully install Stardust and run it. The resulting clusters in the Seurat object,

however, were all NAs. The authors should make an effort to better document how Stardust runs, including the input structure that the tool expects and potential issues that might arise.

Level of Interest

Please indicate how interesting you found the manuscript: Choose an item.

Quality of Written English

Please indicate the quality of language in the manuscript: Choose an item.

Declaration of Competing Interests

Please complete a declaration of competing interests, considering the following questions:

- Have you in the past five years received reimbursements, fees, funding, or salary from an organisation that may in any way gain or lose financially from the publication of this manuscript, either now or in the future?
- Do you hold any stocks or shares in an organisation that may in any way gain or lose financially from the publication of this manuscript, either now or in the future?
- Do you hold or are you currently applying for any patents relating to the content of the manuscript?
- Have you received reimbursements, fees, funding, or salary from an organization that holds or has applied for patents relating to the content of the manuscript?
- Do you have any other financial competing interests?
- Do you have any non-financial competing interests in relation to this paper?

If you can answer no to all of the above, write 'I declare that I have no competing interests' below. If your reply is yes to any, please give details below.

I declare that I have no competing interests

I agree to the open peer review policy of the journal. I understand that my name will be included on my report to the authors and, if the manuscript is accepted for publication, my named report including any attachments I upload will be posted on the website along with the authors' responses. I agree for my report to be made available under an Open Access Creative Commons CC-BY license (http://creativecommons.org/licenses/by/4.0/). I understand that any comments which I do not wish to be included in my named report can be included as confidential comments to the editors, which will not be published.

Choose an item.

To further support our reviewers, we have joined with Publons, where you can gain additional credit to further highlight your hard work (see: https://publons.com/journal/530/gigascience). On publication of this paper, your review will be automatically added to Publons, you can then choose whether or not to claim your Publons credit. I understand this statement.

Yes Choose an item.
