# Supplementary material for: Stardust: improving spatial transcriptomics data analysis through space-aware modularity optimization-based clustering
Source: Gigascience. 2022 Aug 10;11:giac075. doi: 10.1093/gigascience/giac075 (PMC9364686; doi:10.1093/gigascience/giac075)
Supplement: giac075_Reviewer_2_Report_Original_Submission — Quan Nguyen -- 1/27/2022 Reviewed [file giac075_reviewer_2_report_original_submission.pdf]

Reviewer Report

Title: Stardust: improving spatial transcriptomics data analysis through space aware modularity optimization based clustering.

Version: Original Submission **Date:** 1/27/2022

Reviewer name: Quan Nguyen

Reviewer Comments to Author:

This work presents a new clustering method, Stardust, that has the potential to improve stability of clustering results against parameter changing. Stardust can assess the contribution to the clustering result by spatial information relative to gene expression information. Stardust appears to performs better than other methods in the two metrics used in this paper, stability and coefficient of variation. The essence of the method is the use of a spatial transcriptomics (ST) distance matrix as a simple linear combination of physical distance (S) and transcriptional distance (T) matrices. A weight factor is used for the S matrix to control and evaluate the contribution of the spatial information. The effort for evaluating multiple parameters and comparing with several latest methods and across a number of public spatial datasets is a highlight of the work. The authors also made the code available.

Major comments:

- The concept of combining spatial location and gene expression is not new and has been applied in most spatial clustering methods. It is not clear what are the new additions to current available methods, except for a feature to weigh the contribution of spatial components to clustering results.
- The approach to assess the contribution of spatial information, by varying the weight factor from 0 to 1 is rather simple, because the contribution can be nonlinear and vary between spots/cells (e.g. spatial distance becomes more important for spots/cells that are nearer to each other; some genes are more spatially variable than the others; applying one weight factors for all genes and all spots would miss these variation sources)
- The 5 weight factors 0, 0.25, 0.50, 0.75, and 1 were used. However, this range of parameters provided too few data points to assess the impact of spatial factor. As seen in figures, the 5 data points donot strongly suggest a point where the spatial contribution is maximum/minimum due to large fluctuation of values in the y-axis.
- Although two performance metrics are used (stability and variation), there needs to be an additional metric about how the clustering results represent biological ground truth cell type composition or tissue architecture (for example, by comparing to pathological annotation). Consequently, it is unclear if the stardust results are closer to the biological ground truth or not.
- Stardust was tested on multiple 10x Visium datasets, but different types of spatial transcriptomics data like seqFISH, Slideseq, MERFISH, ect. are also common. Extended assessment of potential applications to other technologies would be useful.

Minor comments:

- The paragraphs and figure legends in the Result section are repetitive.
- The result section is descriptive and there is no Discussion section.

Level of Interest

Please indicate how interesting you found the manuscript: Choose an item.

Quality of Written English

Please indicate the quality of language in the manuscript: Choose an item.

Declaration of Competing Interests

Please complete a declaration of competing interests, considering the following questions:

- Have you in the past five years received reimbursements, fees, funding, or salary from an organisation that may in any way gain or lose financially from the publication of this manuscript, either now or in the future?
- Do you hold any stocks or shares in an organisation that may in any way gain or lose financially from the publication of this manuscript, either now or in the future?
- Do you hold or are you currently applying for any patents relating to the content of the manuscript?
- Have you received reimbursements, fees, funding, or salary from an organization that holds or has applied for patents relating to the content of the manuscript?
- Do you have any other financial competing interests?
- Do you have any non-financial competing interests in relation to this paper?

If you can answer no to all of the above, write 'I declare that I have no competing interests' below. If your reply is yes to any, please give details below.

I declare that I have no competing interests

I agree to the open peer review policy of the journal. I understand that my name will be included on my report to the authors and, if the manuscript is accepted for publication, my named report including any attachments I upload will be posted on the website along with the authors' responses. I agree for my report to be made available under an Open Access Creative Commons CC-BY license (http://creativecommons.org/licenses/by/4.0/). I understand that any comments which I do not wish to be included in my named report can be included as confidential comments to the editors, which will not be published.

Choose an item.

To further support our reviewers, we have joined with Publons, where you can gain additional credit to further highlight your hard work (see: https://publons.com/journal/530/gigascience). On publication of

this paper, your review will be automatically added to Publons, you can then choose whether or not to claim your Publons credit. I understand this statement.

Yes Choose an item.
